# Critical analysis of instruments for assessment of presenteeism: an integrative review

**DOI:** 10.47626/1679-4435-2025-1231

**Published:** 2025-12-19

**Authors:** Beatriz Machado de Campos Corrêa Silva, Sergio Roberto de Lucca

**Affiliations:** 1 Universidade Estadual de Campinas, Faculdade de Ciências Médicas, Departamento de Saúde Coletiva, Campinas, SP, Brazil

**Keywords:** presenteeism, evaluation of research programs and tools, occupational health., presenteísmo, avaliação de programas e instrumentos de pesquisa, saúde ocupacional.

## Abstract

Presenteeism has been extensively investigated over the past decades. In the context of new forms of work organization and high competitiveness, the intensification of job demands can lead to insecurity and fear, compromising workers’ well-being, health, and performance, with repercussions for presenteeism, illness, and absenteeism. Considering the conceptual evolution of the topic and its multidimensional causes, this integrative review aimed to describe, analyze, and discuss the instruments available in the literature for measuring presenteeism, focusing on the last 20 years of research. The review followed the Preferred Reporting Items for Systematic Reviews and Meta-Analyses. Based on the findings, the results indicate that the choice of instrument should be made cautiously by occupational health professionals and managers. Future studies should prioritize the development and validation of culturally adapted measures that account for the specificities of different populations and work contexts.

## Abstract (pt)

O presenteísmo tem sido amplamente investigado nas últimas décadas. No contexto de novas formas de organização do trabalho e de elevada competitividade, a intensificação das exigências laborais pode gerar insegurança e medo, comprometendo o bem-estar, a saúde e o desempenho dos trabalhadores, com repercussões em presenteísmo, adoecimento e absenteísmo. Considerando a evolução conceitual do tema e suas causas multidimensionais, esta revisão integrativa teve como objetivo descrever, analisar e discutir os instrumentos disponíveis na literatura para mensuração do presenteísmo, com foco nos últimos 20 anos de pesquisa. A revisão seguiu os Preferred Reporting Items for Systematic Reviews and Meta-Analyses. Com base nos achados, os resultados indicam que a escolha do instrumento deve ser feita com cautela por profissionais de saúde ocupacional e gestores. Estudos futuros devem priorizar o desenvolvimento e a validação de medidas culturalmente adaptadas que considerem as especificidades de diferentes populações e contextos de trabalho.

## INTRODUCTION

In a challenging and uncertain economic environment, the logic of “producing more with less” drives organizations to seek productivity gains by intensifying work demands and imposing increasingly demanding targets. In this context, new forms of organization and management, intensive use of technology, and individual performance and competitiveness evaluations generate insecurity and fear, compromising workers’ well-being, health, and performance. These conditions manifest as both sickness absenteeism and presenteeism.

In Europe, more than two-thirds of workers have reported working despite not feeling physically or emotionally fit.^1-4^ The behavior of attending work despite health limitations is known as presenteeism^5,6^ and is associated with psychosocial, organizational, and structural factors, resulting in impaired concentration, performance, and work quality.^7-12^ Thus, an appropriate operational definition of presenteeism is being at work while perceiving limitations that reduce one’s labor potential.^13^

Several studies show that presenteeism not only reduces productivity but also contributes to the progressive deterioration of health^14-16^ and quality of life.^17,18^ The literature also links this behavior to burnout,^16-18^ musculoskeletal disorders,^19,20^ depression,^21,22^ and an increased risk of cardiovascular diseases.^23,24^

Longitudinal evidence indicates that the frequency of presenteeism is related to both the occurrence and duration of absenteeism.^2,4,25,26^ When recurrent, presenteeism can predict future work incapacity.^4,27^ Longitudinal studies have investigated the number of days participants attended work with impaired activity, classifying presenteeism as short-term (1-2 days), medium-term (3-7 days), and long-term (≥ 8 days per year).^4,28^ Long-term presenteeism is more prevalent among workers facing job insecurity, those working ≥ 50 hours per week, and self-employed workers. It is also more common among groups exposed to organizational or physical demands with adverse health effects. Consequently, long-term presenteeism may better predict severe health problems than short-term presenteeism and shows a stronger association with high levels of psychological distress than with moderate levels.^28^

Some studies discuss possible positive outcomes of presenteeism during professional rehabilitation when the work environment is favorable and socially supportive,^4,27^ such as maintaining productivity and enhancing self-esteem.^11^ In such cases, being present at work reflects motivations linked to the work environment that foster continuity and a positive interpretation of attendance. However, this perspective is rarely addressed in research.

To estimate the prevalence and impact of presenteeism across different occupational groups, several instruments have been developed over the past two decades. Most consist of questionnaires covering dimensions associated with presenteeism, such as physical and mental illness, work context, psychosocial and organizational factors, and productivity outcomes. The items, typically self-reported on Likert-type scales, should display adequate specificity and sensitivity to the construct and demonstrate sound psychometric properties.

Because of the conceptual evolution of the phenomenon and its multidimensional causes in a constantly changing work environment, this integrative review aims to describe, analyze, and discuss the instruments available in the literature to measure presenteeism. The topic is relevant because diagnosing presenteeism and identifying its triggering factors can support preventive actions within organizations and guide the promotion of healthier work environments that protect workers’ health.

## METHODS

We conducted a scoping review of the literature on presenteeism and assessment instruments, encompassing different study designs with an emphasis on practical applicability.^29,30^ The aim was not to judge evidence quality, but to map the main self-report instruments used in research whose measurement properties assess productivity loss associated with compromised worker health.

The review followed the methodological framework proposed by JBI, adapted to the study’s objectives. The process involved i) defining the research question and aim; ii) identifying relevant studies to ensure breadth and scope; iii) selecting studies according to predefined criteria; iv) charting data; v) synthesizing the results in relation to the aim and question; and vi) presenting the findings, identifying implications for concept, practice, and research.

The guiding question was: what instruments exist and are used to identify presenteeism? We then defined keywords drawn from Medical Subject Headings capable of retrieving articles pertinent to the topic, namely “translational medical research” and “knowledge translation.”

Searches for relevant studies were performed in MEDLINE, PsycInfo, SciELO, PubMed, LILACS, the Virtual Health Library, and ePROVIDE™. The search strategy was developed by the first author in collaboration with a librarian from the School of Medicine at the State University of Campinas. We included articles published in health journals indexed in the last 20 years. Searches were carried out between January and June 2022, combining terms related to the construct of interest with the descriptors “presenteísmo” and “perda de produtividade,” linked by the Boolean operators “OR” and “AND” as well as “instrumentos” and “mensuração” (genérico AND autorrelato) and their English equivalents.

Eligibility criteria included i) instruments validated for psychometric properties; ii) measurement of health-related changes in productivity; iii) a generic scope, not restricted to a specific condition; iv) assessment of productivity from the respondent’s perspective (worker self-report); v) an estimate of the extent to which perceived health status affected job performance; and vi) availability of original, full-text articles published in English or Portuguese. We excluded instruments that did not include at least 20% of items on presenteeism, that were 20 years old or more since publication, or that lacked reported or evaluated psychometric information.

**Figure 1:**
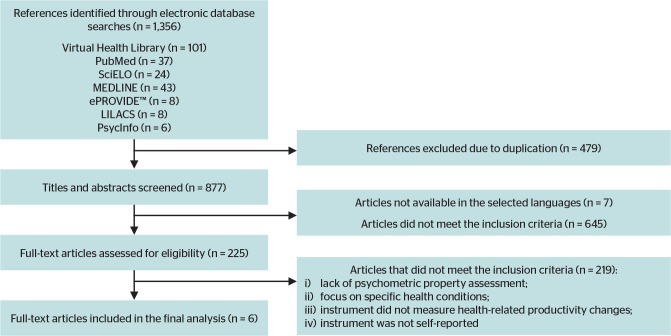
Flowchart of the literature review.

Study selection occurred in successive stages: i) title and abstract screening; ii) application of inclusion and exclusion criteria; iii) definition and extraction of the information of interest, followed by analysis; and iv) retrieval and full-text reading of the selected articles. Figure 1 presents the review flowchart according to the pre-established criteria.

## RESULTS

Eligible studies were independently classified by the authors. After reading each article, data were summarized using a standardized form that included the instrument’s name and acronym, authors, year of publication, country of origin, language, total number of items, number of items specifically related to presenteeism, and recall period.

Six generic instruments applicable to any economically active population were identified. The selected instruments were published between 2002 and 2019, with two originating from the United States, two from Canada, one from Japan, and one from Brazil. Table 1 presents the included instruments, summarizing the main information and providing a brief description of their purpose, total number of items, items specific to presenteeism, and recall period.

**Table 1:** Description of generic instruments measuring presenteeism

Instrument	Authors/year and country of origin	What the instrument assesses and its purpose	Number of items		Recall period
			Total	Presenteeism	
SPS-6	Koopman et al. (2002)^31^ USA	Assesses the impact of presenteeism and presenteeism-related behavior resulting from decreased concentration and performance capacity	6	6	4 weeks
HRPQ-D	Kumar et al. (2003)^32^ USA	Measures the impact of illness on productivity at work and in domestic tasks	9	4	Daily
EQCOTESST	Vézina et al. (2011)^33^ Canada	Evaluates musculoskeletal disorders, occupational accidents, and mental health in the workplace as well as general health and potential productivity loss	142	30	12 months
VOLP	Zhang et al., (2011)^34^ Canada	Analyzes the impact of health conditions on monetary productivity loss, employment status, absenteeism, presenteeism, unpaid work, availability of substitutes, and compensation mechanisms	37	7	1 week
IAPT	Menezes & Xavier (2017)^35^ Brazil	Assesses productive performance during the workday and the impact of health conditions	10	6	Daily (every 2 hours)
SPQ	Muramatsu et al. (2019)^36^ Japan	Measures pure presenteeism through self-reporting of the impact of health problems on productivity and individual performance	1	1	4 weeks

EQCOTESST = Québec Survey on Working and Employment Conditions and Occupational Health and Safety; HRPQ-D = Health-Related Productivity Questionnaire - Diary; IAPT = *Instrumento Rápido para Avaliação Subjetiva de Produtividade Laboral Intrajornada*; SPQ = Single-Item Presenteeism Question; SPS-6 = Stanford Presenteeism Scale, 6-item version; VOLP = Valuation of Lost Productivity.

Many studies failed to report essential information on several aspects of methodological validation, such as test-retest procedures, interand intra-rater reliability, measurement error, hypothesis testing, structural or factor validity, cross-cultural validity, and responsiveness. We chose not to assess methodological quality because the techniques used to analyze validity and reliability varied substantially across studies, limiting comparability and preventing us from indicating which instrument would be most appropriate for identifying and evaluating presenteeism. Table 2 presents a summary of the measurement properties identified and the populations in which they were established.

**Table 2:** Studies on the psychometric properties of presenteeism instruments

Instrument	Study (authors, year)	Objective	Study population	Psychometric properties assessed	Main findings
SPS-6	Cicolini et al. (2016)^37^	Verify validity and reliability of the Italian version of the SPS-6	Nursing workers (n = 229)	Validity; reliability; cross-cultural validity	Internal consistency (α = 0.72); external validity; factor analysis explaining 71.2% of the variance
	Paschoalin et al. (2012)^22^	Adaptation and validation for Brazilian Portuguese	Nursing workers (n = 229)	Validity; reliability; cross-cultural validity	Test-retest (weighted κ, 0.61-0.94); temporal stability (ICC = 0.91); internal consistency (α = 0.70)
	Ferreira et al. (2010)^38^	Validation in Portuguese of the WLQ and SPS-6 and presentation of metric properties	Education and health workers (n = 305)	Validity; cross-cultural validity	Internal consistency (α = 0.780); item-total correlation; overall α = 0.815
	Koopman et al. (2002)^31^	Development and testing of the SPS-6	Health workers (n = 164)	Validity; reliability	Internal consistency (α = 0.80); construct validity (factor analysis, varimax rotation with Kaiser normalization); concurrent validity: productive time (*r*_s_ = 0.53; p < 0.001) and work accomplished (*r*_s_= 0.47; p < 0.001); criterion validity: work-related disability (mean, 21.0; SD, 3.9) vs. no disability (mean, 23.5; SD, 3.8; *t*[159] = 3.54; p < 0.001); discriminant validity: job satisfaction (*r*_s_= 0.15; p < 0.05) and job stress (*r*_s_= 0.22; p < 0.01); correlation with SPS-32
HRPQ-D	Kumar et al. (2003)^32^	Validate a generic productivity measure (HRPQ-D)	Adolescents with infectious mononucleosis (n = 42)	Validity; responsiveness	Construct validity (Pearson correlation); responsiveness (Pearson correlation)
EQCOTESST	Vézina et al. (2011)^33^	Understand relationships between work organization, health, and safety; identify hazardous work conditions and describe consequences	Workers from several sectors (n = 5,071)	Reliability	Items adapted from the Job Content Questionnaire (α = 0.81) and the Copenhagen Psychosocial Questionnaire (α = 0.83)
VOLP	Gelfand et al. (2021)^39^	Adapt the questionnaire to a caregiver version and assess feasibility/validity of online administration (VOLP-caregiver)	Caregivers (n = 383)	Validity; reliability	Reported evidence of validity and reliability
	Zhang et al. (2011)^34^	Develop and validate a tool to measure productivity loss in monetary units	Participants with rheumatoid arthritis (n = 152)	Validity; reliability	Reported evidence of validity and reliability
IAPT	Menezes & Xavier (2017)^35^	Develop, validate, and test clarity and reliability of the rapid instrument for intraday assessment of productivity	Sample not reported	Validity; reliability	Relevance, 9.11 ± 0.93; clarity, 9.23 ± 0.75; split-half (r^2^ = 0.78); α = 0.91 (managerial variables) and α = 0.80 (physical and mental variables); convergent validity: Pearson correlation with HPQ (r^2^ = 0.86) and reliability with HLQ (r^2^ = 0.82)

EQCOTESST = Québec Survey on Working and Employment Conditions and Occupational Health and Safety; HLQ = Health and Labor Questionnaire; HPQ = Health and Work Performance Questionnaire; HRPQ-D = Health-Related Productivity Questionnaire - Diary; IAPT = *Instrumento Rápido para Avaliação Subjetiva de Produtividade Laboral Intrajornada*; ICC = intraclass correlation coefficient; SD = standard deviation; SPS-6 = Stanford Presenteeism Scale, 6-item version; SPS-32 = SPS, 32-item version; VOLP = Valuation of Lost Productivity; WLQ = Work Limitations Questionnaire.

The Stanford Presenteeism Scale, 6-item version (SPS-6), indirectly measures cognitive, behavioral, and emotional aspects related to work. The instrument contains six items distributed across two dimensions: three address physical aspects (work completed) and three address psychological aspects (ability to concentrate).^31,37,38^ The study developed by Koopman et al. (2002)^31^ demonstrated good psychometric properties, with strong evidence for most measurement domains, including content validity, internal consistency, construct validity, convergent validity, and responsiveness.^17^ The Italian version of the SPS-6 also demonstrated the instrument’s reliability. In the cross-cultural adaptation and validation of the SPS-6 for Brazilian Portuguese, Paschoalin et al. (2012)^22^ found good psychometric properties comparable to those described in international studies.^40,41^

The Health-Related Productivity Questionnaire - Diary (HRPQ-D), in its short version, evaluates daily sickness absenteeism and presenteeism as well as measuring productivity loss from an economic perspective. The validation study conducted by Kumar et al. (2003)^32^ demonstrated good construct validity and reasonable responsiveness, possibly influenced by the small sample size and its composition (participants aged 14-32 years).^17^ Although concise, the HRPQ-D does not account for potential compensation mechanisms or team dynamics.^40^ No cross-cultural adaptation or validation studies for Portuguese were found.

The Québec Survey on Working and Employment Conditions and Occupational Health and Safety (EQCOTESST) was developed for use among both formal and informal workers with varying levels of qualification across multiple economic sectors. It is a multifactorial instrument composed of items relevant to workers’ health. The presenteeism questions were adapted from the Job Content Questionnaire and the Copenhagen Psychosocial Questionnaire, distributed across different domains of the instrument and later measured to assess the impact of these behaviors on workers’ health and their possible causes,^33^ thus providing a broader understanding of presenteeism.

Studies using the EQCOTESST showed that 53% of participants reported presenteeism behavior in the previous 12 months. Most experienced short-term presenteeism (fewer than 10 days), while a smaller proportion experienced long-term presenteeism.^28^ Long-term presenteeism was more frequent among those working 50 or more hours per week, self-employed workers, and individuals exposed to organizational or physical demands with negative health effects. It was also associated with higher levels of psychological distress.^6,33^ The main disadvantages of the EQCOTESST are its large number of items and lengthy recall period. The instrument has not yet been translated or validated for Brazilian Portuguese.

The Valuation of Lost Productivity (VOLP) is an instrument that assesses monetary productivity loss at work due to health problems and covers six aspects: employment status, job characteristics, absenteeism, work performance, unpaid work, and work environment. Productivity loss is estimated solely from internally collected data.^34^ Although evidence supports its validity for measuring productivity loss among individuals with rheumatoid arthritis at different levels of functional impairment, the main drawbacks of VOLP are its large number of items and inclusion of content not directly related to presenteeism. The VOLP has not yet been translated or validated for Portuguese.

The Brazilian instrument *Instrumento Rápido para Avaliação Subjetiva de Produtividade Laboral Intrajornada*, developed by Menezes and Xavier (2017),^35^ was designed specifically to measure productivity during the workday and its variations caused by presenteeism. The questionnaire includes aspects of physical, mental, and occupational health at short intervals, thereby reducing recall bias. In the authors’ study, it demonstrated satisfactory psychometric properties. Despite its quick application, it may face resistance for requiring frequent work interruptions.

The Single-Item Presenteeism Question (SPQ) measures absolute presenteeism based on a single question: “On a scale from 1% to 100%, where 100% represents the best performance you could achieve at your job if you were not limited by illness or injury, how would you rate your overall work performance on the days you worked during the past four weeks (28 days)?” This question refers to self-perceived performance, considering the effects of any illness or injury, and allows estimation of the economic cost of presenteeism. In patients with low back pain, the study conducted by Kigozi (2021)^42^ showed moderate to strong correlations between presenteeism, pain, disability, and physical dimensions, as well as high responsiveness to changes in productivity-presenteeism scores. Similar results were reported by Muramatsu et al. (2021)^36^ among smalland medium-sized enterprise workers, confirming the construct validity and responsiveness of SPQ. The Persian version, evaluated by Khanmohammadi et al. (2018)^43^ in patients with low back pain, demonstrated good reliability. As a recent instrument, few studies are available. Its main limitation is the use of a single question, which may simultaneously capture personal attributes and work environment factors. Additional research is needed to strengthen the psychometric evidence of this instrument (Table 3).

**Table 3:** Analysis of the advantages and disadvantages of presenteeism instruments

Instrument	Advantages	Disadvantages
SPS-6	Assesses presenteeism exclusively; short instrument; widely tested; short recall period	Lack of completeness and clarity in some items, which may compromise the instrument’s comprehensibility (Tang; Beaton)
HRPQ-D	Assesses productivity loss due to absenteeism and presenteeism; estimates productivity loss costs; short recall period	Does not evaluate potential compensation mechanisms or team dynamics (Tang); only one psychometric study available
EQCOTESST	Composed of multifactorial items that assess workers’ health habits and conditions	Lengthy instrument; long recall period
VOLP	Assesses productivity loss due to absenteeism and presenteeism; short recall period; can be applied in modules	Lengthy if all modules are administered together; few psychometric studies conducted among workers with other health conditions
IAPT	Assesses presenteeism exclusively; short instrument; short recall period	Requires more psychometric studies and validation in different populations
SPQ	Assesses presenteeism exclusively; short instrument; short recall period; allows estimation of costs of productivity loss	Few psychometric studies available; requires validation in other population

EQCOTESST = Québec Survey on Working and Employment Conditions and Occupational Health and Safety; HRPQ-D = Health Related Productivity Questionnaire - Diary; IAPT = *Instrumento Rápido para Avaliação Subjetiva de Produtividade Laboral Intrajornada*; SPQ = Single-Item Presenteeism Question; SPS-6 = Stanford Presenteeism Scale, 6-item version; VOLP = Valuation of Lost Productivity.

## DISCUSSION

Regarding the objectives of the instruments analyzed, two main approaches were identified: one focused on evaluating the relationship between physical, mental, or emotional problems or symptoms and presenteeism, considering its detrimental effects on workers’ health^1,33^; and another aimed at measuring the cost of productivity loss resulting from health-related presenteeism that compromises work performance. The number of specific items related to presenteeism varied widely among the instruments, ranging from 1 to 30.

A substantial heterogeneity in sample size was also observed, which compromises comparability among studies. In health research, sample size is an important factor, as its adequacy ensures the statistical validity of the results.^44^ The representativeness of the sample is essential for the reliability of findings: the larger the number of participants, the more accurate the results tend to be, and the smaller the sampling error.^44^

The validity and statistical significance of an instrument reflect the degree of confidence that the results obtained accurately represent the target population.^45,46^ From this perspective, classical psychometrics considers an adequately sized sample sufficient to assess representativeness, whereas modern psychometrics defines sample size as one of the validity criteria for an instrument. To ensure adequacy, it is recommended that studies include a minimum of 500 participants, while high-quality instruments should be based on samples with at least 1,000 respondents.^47^ In view of its importance, future research should aim to include as many participants as possible.

Evaluating the psychometric properties of self-report instruments that measure subjective constructs is a fundamental step prior to investigating associations between these instruments and health outcomes, particularly when applied to populations different from those in which they were originally developed.^41,48^

The quality of an instrument is determined by psychometric variables that demonstrate its reliability and validity.^49^ Reliability refers to the ability to reproduce consistent results over time and across contexts. A measure can be reliable without being valid; however, the opposite is not true. The absence of reliability directly undermines validity. The most common techniques used to assess reliability are the test-retest and split-half methods. Validity, in turn, refers to an instrument’s ability to measure precisely what it intends to measure. Evidence of validity can be verified according to the trinitarian model, which comprises content validity, criterion validity, and construct validity.^50-52^

Other psychometric properties have attracted increasing interest, such as responsiveness and cross-cultural adaptation. Responsiveness is defined as the ability of an instrument to detect changes in functionality, health status, or an individual’s perception over time.^53^ Cross-cultural adaptation, on the other hand, ensures that instruments designed to assess subjective constructs maintain their validity and reliability properties when applied in different cultural contexts.^53^

The instruments included in this review were evaluated, to varying degrees, across three core domains of psychometric quality: reliability, validity, and responsiveness. Although all are of a generic nature, many studies focused on specific health conditions. To ensure the psychometric consistency of an instrument, it is essential to demonstrate both its reliability and validity, thereby confirming its applicability in different contexts. Instruments designed for broad use should be tested and retested in diverse environments so that their psychometric properties can be generalized.^41^

However, several limitations should be considered. Despite their wide use in research on health, organizational behavior, and economics, self-report instruments are subject to multiple cognitive and socio-motivational biases.^54^ Such biases may produce inaccurate association estimates and lead to the overestimation or underestimation of risk parameters in epidemiological studies.^55^ The main biases include comprehension and interpretation bias, related to how respondents understand the questions; response bias, which occurs when participants attempt to preserve their self-image or fear their answers may be identified by supervisors; and recall bias, influenced by the recall period, the characteristics of the construct being assessed, the profile of the sample (eg, age), and the study design.^54-56^

There was also considerable variation in the recall periods used by the instruments (ranging from daily to 12 months), which may affect response accuracy. Studies indicate an inverse relationship between recall period length and data precision: longer periods increase the likelihood of memory lapses, whereas shorter ones may fail to capture relevant events adequately.^57^ Although no consensus exists regarding the ideal interval, shorter recall periods tend to minimize recall bias in self-reported measures.^56,58,59^

The main strategy to mitigate self-report bias is for researchers to be familiar with an instrument’s psychometric properties before its application, particularly its reliability and validity, to ensure the quality of the results obtained.^54,57^

Another important aspect concerns the scarcity of cross-cultural validation studies. The purpose of this process is to adapt an instrument so that it is culturally comprehensible while preserving the meaning and intent of the original items. Adaptation should follow rigorous methodological guidelines to ensure validity, reliability, and conceptual equivalence across cultures and populations.^60^

Overall, the instruments analyzed in this review demonstrated good psychometric properties. However, before selecting an instrument, researchers should consider the available evidence regarding its reliability and validity, the study objectives, the construct to be measured, the type of organization, and the target population.^14,17^

This review has limitations, including the inclusion of articles published only in Portuguese and English, which may have led to the exclusion of relevant studies published in other languages.

## CONCLUSIONS

Based on this review’s findings, it is concluded that self-report instruments are susceptible to bias. Future instruments should aim to minimize these biases by considering, among other factors, the duration of the recall period, the nature of the health condition investigated (acute or chronic), the relationship between illness and occupation or individual characteristics, the sample profile, and the self-report approach itself, ideally incorporating peer assessments as well.^57^

Available evidence on the psychometric properties of generic self-report instruments measuring health-related presenteeism indicates adequate reliability and validity.^55^ However, most studies have focused on identifying the determinants of presenteeism to detect it solely in relation to illness. Psychosocial work-related factors, which are strongly correlated with presenteeism, should also be considered. Among the instruments analyzed, EQCOTESST is the only one encompassing both changes in health status and psychosocial dimensions.

This review identifies opportunities for future investigations to explore whether illness development stems from work, organizational factors, or individual characteristics, not merely how illness affects performance, productivity, and economic outcomes. The goal was to analyze and reflect on gaps in generic self-report instruments for measuring presenteeism rather than to assess the methodological quality of the included studies in depth.

Presenteeism remains a current and relevant topic, with considerable research potential to improve its identification and prevent the worsening of health conditions among workers. Advancements in this field may help clarify morbidities or situations of discomfort and maladjustment at work, reduce absenteeism, and guide preventive actions, as well as the identification and reflection on organizational stressors.
